# Analysis of a Trichinellosis Outbreak in Poland after Consumption of Sausage Made of Wild Boar Meat

**DOI:** 10.3390/jcm11030485

**Published:** 2022-01-18

**Authors:** Mirosław Różycki, Weronika Korpysa-Dzirba, Aneta Bełcik, Tomasz Pelec, Justyna Mazurek, Tomasz Cencek

**Affiliations:** 1 Department of Parasitology and Invasive Diseases, National Veterinary Research Institute, Partyzantow Avenue 57, 24-100 Pulawy, Poland; mrozycki@piwet.pulawy.pl (M.R.); aneta.belcik@piwet.pulawy.pl (A.B.); tcencek@piwet.pulawy.pl (T.C.); 2 Department of Internal Medicine and Diagnostics, Faculty of Veterinary Medicine and Animal Sciences, Poznan University of Life Sciences, 60-637 Poznan, Poland; tomaszpelec.wet@gmail.com; 3 Department of Epidemiology, Voivodship State Sanitary-Epidemiological Station in Poznan, 61-705 Poznan, Poland; epidemiologia@wssepoznan.pl

**Keywords:** *Trichinella* spp., trichinellosis, wild boar, sausage, outbreak

## Abstract

An outbreak of trichinellosis due to the consumption of sausage made from wild boar meat unexamined for the presence of *Trichinella* spp. was reported in Poland in December 2020. The outbreak affected eight people. Examination of the sausages made of wild boar meat collected during epidemiological investigation indicated a high level of *Trichinella* spp. Larvae per gram (>30 lpg) and therefore the threat of an infection in humans after consumption of such product was significant. Over the years, the main source of trichinellosis in Poland has been wild boar meat, and the majority of trichinellosis cases were related to the consumption of traditional raw meat products such as Polish sausage. Taking this into account, there is the need for better education of consumers in the *Trichinella* spp. endemic regions and among cultures consuming traditional raw meat products.

## 1. Introduction

Each year, several cases of trichinellosis are reported in Poland [1,2,3,4,5]. Diagnosis of this disease is made in patients presenting signs and symptoms typical for trichinellosis, positive laboratory test for *Trichinella* spp., and consumption history of potentially infective food with special attention to meat products eaten raw or undercooked. Symptoms of trichinellosis may vary from mild to severe illness [6]. First signs of the disease may occur 1–3 days after consumption of infected meat (enteral phase) [7]. They are usually mild and manifest by abdominal pain, diarrhea, vomiting, and sometimes, fever. At this stage, diagnosis is difficult since the symptoms are unspecific or similar to those of other diseases caused by virus infection or food intoxication. After one or two weeks, the typical signs for *Trichinella* spp. infection appear and can include fever, headache, myalgia, fatigue, and face edema. They can last for a few weeks (parenteral phase) and in some cases may be prolonged. Laboratory diagnosis is based on immunoserological methods, mainly ELISA test, with the use of the excretory/secretory antigen. ELISA is the most commonly used serological method to detect *Trichinella* infection in humans and animals [8,9]. Effectiveness of mebendazole is highly dependent on administration time, with the most effective treatment being observed at early stages of infection [10]. The treatment is focused on eliminating adult forms of nematodes from the intestines. The bioavailability of drugs commonly used against human trichinellosis is limited for muscle-stage larvae. Consequently, in a later phase of infection, the treatment must be prolongated. The use of antihelmintic therapy may be ineffective in chronical phase, and antiparasitic treatment introduced in advanced stage is often combined with steroids [7].

According to the annual reports on “Infectious diseases and poisonings in Poland” issued by the Polish National Public Health Institute, between 2016 and 2020, a total of 37 cases of trichinellosis were registered, which indicates an average incidence of 0.02 cases per 100,000 people. Nearly half (48.6%) of these cases were noted in Wielkopolskie Voivodeship, located in the west part of Poland [1,2,3,4,5]. One of the parts of this region is Kościan District [11]. The forest area in this district consists of 7665.3 km^2^, and the density of wild boars is 1.3 animals per km^2^. This area is dominated by coniferous forest, with the majority being pines (68%), but there are also black alder (11%) and oak (8%) trees. Due to the significant share of pine forests, this area is characterized by a shortage of protein available to animals. Depending on the season, the wild boars feed on undergrowth fruit, crop plants, invertebrates, small rodents, and carrion, which can be a reservoir for *Trichinella* spp. [12]. It may be possible that offal left in the hunting grounds may also be a source of infection for carnivores, scavengers, and omnivores, including wild boars.

Culling wild animals is planned and coordinated by hunting clubs and game breeding centers. Annual plans assume the acquisition of large game such as wild boar, roe deer, red deer, mouflon, and fallow deer as well as small game. According to the annual hunting plan, the wild boar acquisition in the 2020/2021 season in the hunting districts located partly and entirely within the Kościan Forest District amounted to 859 animals. However, due to African swine fever risk reduction, provisions for additional hunting were also made (295 animals in 2020).

Within 2011–2020, 129 cases of human trichinellosis were diagnosed. Three voievodships: Kuyavia, Wielkopolska, and West Pomerania participated with more than half (51.9%) of all cases, and the number of human cases in these regions were 32 (24.8%), 26 (20.2%), and 9 (7%), respectively [1,2,3,4,13,14,15,16,17,18]. The *Trichinella* spp. prevalence in wild boars in these regions reaches up to 0.26% [19].

## 2. Materials and Methods

### 2.1. Outbreak Investigation

At the end of December 2020, the District Sanitary Inspector was informed by the primary health care doctor from Koscian (Wielkopolskie voivodeship) about three patients who had clinical symptoms indicating a potential infection caused by *Trichinella* spp. The district veterinarian, upon notification of the suspicion of trichinellosis, undertook actions in order to detect the source of infection. The procedure in such cases was established based on a contingency plan set by Chief Veterinary Officer according to EU regulation 1375/2015 for cases of *Trichinella* spp. detection in farm and wild animals in Poland [20]. According to the contingency plan, the main actions are aimed at controlling the following elements:

(1) Identification of infected carcasses and parts thereof containing muscle tissue;
(2) Handling of infected carcasses and parts thereof;
(3) Search of the sources of infection and its spread among fauna;
(4) Determination of the species of detected *Trichinella* spp.

Samples (see Section 2.3) collected by the Veterinary Inspector for epizootic purposes were transferred to the National Reference Laboratory for Trichinellosis (NRL) [21].

### 2.2. Epidemiolological Investigation

Epidemiological investigation was performed in cooperation with the Sanitary Inspection Service in Poznań and Veterinary Inspection and consisted of collecting information from all individuals involved in the outbreak and taking suspected samples for further analysis. The epidemiological information was collected with use of ZLK-1 questionnaire “ZLK-1 Notification of suspicion or diagnosis, infection or infectious disease”. This form was supplemented with information on the occurrence of diarrhea, fever, myalgia, facial edema, subconjunctival and subungual extravasation, and eosinophilia. Moreover, where it was possible, the information about the results of serological tests was included. 

According to Commission Decision (EU) 2018/945, the following criteria were applied:

1. Clinical criteria: any person with at least three of the following six criteria: fever, muscle pain, diarrhea, swelling of the face, eosinophilia, and subconjunctival, subungual and retinal hemorrhages.
2. Laboratory criteria: at least one of the following two criteria: demonstrating the presence of *Trichinella* larvae in a muscle biopsy, demonstrating the presence of specific antibodies to *Trichinella* (IFA, ELISA, or Western blot).
3. Epidemiological criteria: at least one of the following two epidemiological links: exposure to contaminated food (meat), exposure to the same source.

Cases are classified as possible, probable, and confirmed. A possible case means a case meeting the clinical criteria without epidemiological or laboratory evidence.

Probable case applies to any person meeting the clinical criteria with an epidemiological link. Confirmed case meets clinical and the laboratory criteria [22,23].

### 2.3. Animal Species Identification as Meat Origin

Since the origin of meat was unclear, two samples of equal number of sausages were delivered to the laboratory for species identification. They were transported and stored frozen. After thawing, the sausage casings were removed, and particles of muscle tissue were selected and cleared from fat and spices. Samples were examined for animal species identification with isoelectric focusing on polyacrylamide gels in gradient pH, according to a methodology previously described [24]. Electrophoresis was performed on the PhastSystem apparatus (Amersham, UK) on ultrathin gradient gels (PhastGel IEF) at pH 3–9. This type of electrophoresis is based on the amphoteric characteristic of proteins (depending on the pH of the environment, they can act as either acids or bases). Soluble proteins migrate in an electric field in a pH gradient according to their characteristic pI (isoelectric point). Separated and immobilized proteins were stained and compared with reference material.

### 2.4. Larvae Detection and Identification

Two sausage samples were delivered frozen to the laboratory. After thawing, samples weighing 50 g each were prepared and examined by magnetic stirrer-assisted digestion method [25]. Prior to the digestion, the samples were soaked in water for one hour to remove salts, soften the muscle tissue, and facilitate the digestion process. Sediments were examined under 80× magnification on Trichinoscope FF VII (Ratenov, Germany). Larvae were collected in ethyl alcohol for further molecular identification. The DNA was extracted with IQtm System kit (Promega, Mdison, WI, USA). The PCR reaction was carried out according to the protocol provided by European Reference Laboratory for parasites [26]. For species identification, three sequences belonging to the ITS1, ITS2, and ESV regions were used [27].

## 3. Results

### 3.1. Outbreak Investigation

Trichinellosis was confirmed in eight patients who were family and friends of the hunter who had provided them with the homemade raw Polish sausage. All patients belonged to three families inhabiting the Koscian district. The owner of the wild boar carcass confirmed that the wild boar had been hunted in this area. The epidemiological investigation indicated the source of infection—Polish sausage made from wild boar. People who had consumed the same meat but in the form of steamed sausage did not have any symptoms of *Trichinella* spp. infection.

The summary of the symptoms observed in patients with confirmed trichinellosis is shown in Table 1. The amount of consumed sausage could not be established.

### 3.2. Epidemiological Investigation

The sausages were produced at the beginning of autumn 2020 by the hunter for own consumption; however, they were also distributed among his family and friends. Wild boar meat was used for production of raw Polish sausage. The sausage was medium shredded with natural casings. The product was smoked with cold smoke, and the core temperature did not exceed 35 °C.

The collected data indicated that the suspect sausages consumed by the hunter and his family were also delivered to two related families and friends. As a part of epidemiological investigation, the Koscian Local Veterinary Officer (LVO) collected two samples of suspected raw sausages. During the epidemiological investigation, the hunter firstly declared that the sausages were made of roe deer (*Capreolus capreolus*) meat, and later he claimed that he used wild boar meat that had been tested for the presence of *Trichinella* spp. in the veterinary laboratory in Leszno; however, he could not provide any documentation confirming results of investigation. The laboratory itself confirmed that such samples were not noticed in their documentation. The LVO responsible for the Leszno district was therefore also informed about the trichinellosis outbreak. The hunter informed the Koscian LVO that the sausage had also been distributed to one family inhabiting the Leszno District; however, the contact details for this family could not be established. The examination of suspected sausage samples by digestion method in the laboratory in Czempin (Koscian District) confirmed the presence of *Trichinella* spp. The District Sanitary Inspector and the individuals who consumed the sausage were informed of these results.

The meat species identification confirmed that the sausage was made from wild boar meat.

Samples were examined for *Trichinella* spp. detection. The presence of *Trichinella* spp. in raw Polish sausage was confirmed after the analysis of two samples of Polish sausage using the artificial digestion method [25].

**Figure 1:**
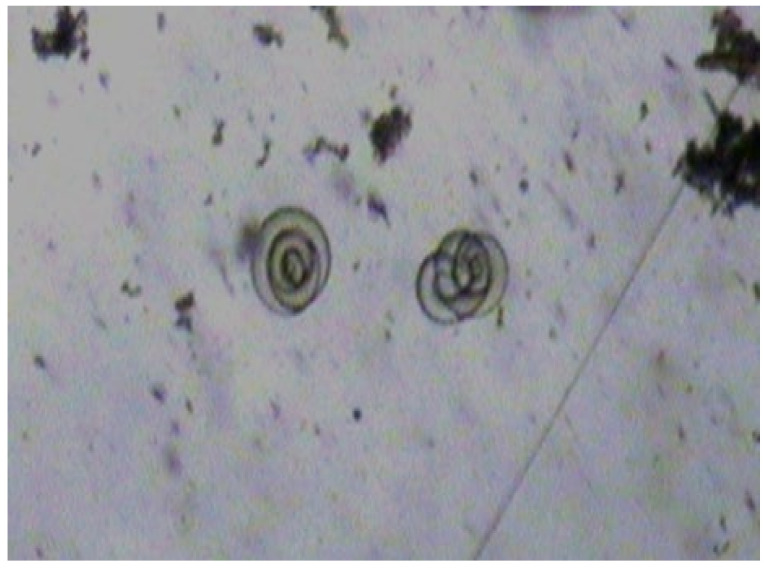
The dead *Trichinella* spp. larvae detected in raw Polish sausage after artificial digestion method.

In the first sample, 34 larvae per gram were detected, and 54 larvae per gram in the second one; all larvae were dead. An example of detected larvae is presented in Figure 1.

**Figure 2:**
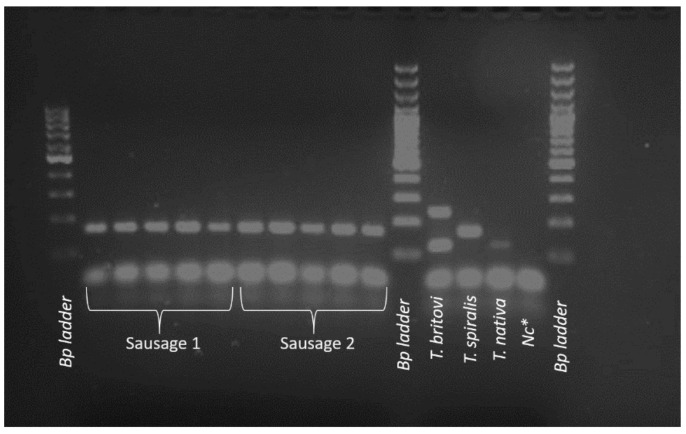
PCR identification of *Trichinella* spp. detected in raw Polish sausage. Samples on patterns marked as Sausage 1 and Sausage 2 (173 bp) have been identified as *T. spiralis*. Positive controls: *T. britovi*, *T. spiralis*, *T. nativa*; Nc*—negative control (H_2_O).

Further analysis using PCR technique according to the EURLP protocol was performed, confirming the presence of *T. spiralis*. The results of this analysis are presented in Figure 2.

## 4. Discussion

In recent years, a significant change in the occurrence of *Trichinella* spp. in the population of farmed and wild animals has been observed. Due to changes in pig farming, such as strengthening of the veterinary supervision over pig production, the role of pigs as a main source of trichinosis in humans has decreased in favor of wild boar meat [28]. Risk reduction, transfer of responsibility for the safety of food products from inspection to the producer, high testing costs, globalization of the market, and the need for price competition on world markets have all fostered the ideas of a new approach to ensure pig meat safety [29]. In December 2004, the Scientific Panel on Biological Hazards (BIOHAZ) of the European Food Safety Authority (EFSA) adopted an opinion on the adequacy and detail of freezing methods allowing human consumption of meat contaminated with *Trichinella* or *Cysticercus*. A year later, in March 2005, BIOHAZ adopted an opinion on the risk assessment of repeated inspection of animals for slaughter in areas with low prevalence of *Trichinella* spp. EFSA concluded that *Trichinella* spp. from the consumption of pig meat poses a moderate risk to public health [30]. Better farming practices, controlled housing conditions, and meat inspection reduced the risk of trichinellosis in the EU. However, the risk still comes from free ranging and poor housing conditions of backyard farming of pigs in small family farm production systems.

In the wildlife environment, the implementation of rabies prevention (increase in the population of foxes), including the Natura 2000 program, aimed to increase the biodiversity; this indirectly led to increasing the number of hosts susceptible to *Trichinella* spp. infection [31]. However, restrictions introduced in some European countries related to taking carcasses from hunting grounds and the utilization of inedible parts of animals as well as higher agricultural education contributed to the reduction of *Trichinella* spp. in the wild animal population. In Poland, the spread of *Trichinella* spp. in the population of wild animals is favored by the behavior of hunters who, after skinning or gutting, leave the carcasses of animals in the hunting grounds.

According to the Commission Implementing Regulation (EU) 2015/1375, carcasses of all animals susceptible for *Trichinella* spp. should be examined for the presence of this parasite [20]. However, epidemiological reports indicate that in spite of these regulations, uninspected meat is still incidentally used for local production of raw meat products. Mainly hunters, along with their family and friends, are those at risk of acquiring trichinellosis after consumption of wild boar meat, especially if the meat has not undergone a proper heat treatment [32]. Sausages made with meat from domestic pigs mixed with contaminated wild boar meat have also been a source of human infections [33]. During the last two decades, such incidents have been reported in many European countries. Wild boar meat acts as source of infection not only for trichinellosis cases but also for other zoonoses [34]. Since 2014, there have been no human cases induced by the consumption of pig meat raised in controlled conditions [35]. On the contrary, there has been an increase in cases caused by the consumption of wild boar meat [36].

In Poland, wild boars are mainly infected with *T. spiralis* (78.31% of infections), followed by *T. britovi* (18.57%) and mixed infections *T. spiralis/T. britovi*—2.73% [37]. The other two species are rare: *T. pseudospiralis* (0.28%) and *T. nativa* (0.03%, only one case) were confirmed in wild boars [38]. The official data based on the presence of *Trichinella* larvae in wild boar muscle tissue (examined with digestion method) indicate the high prevalence (varying from 0.2% to 0.5%). An assessment of the epidemiological situation of trichinellosis in Poland was performed in 2011 by Sadkowska-Todys and Gołąb (2013). According to their analysis, in 2011, there were three trichinellosis outbreaks notified, involving 22 human cases. All three outbreaks, similarly to the one described in this paper, were caused by the consumption of products prepared from wild boar meat, mostly raw sausages, and were limited to the people from single families and close circles of friends [39]. However, a few years prior in 2007, in a north-west region of Poland, a trichinellosis outbreak occurred involving 214 cases, following the consumption of raw meat sausages. Molecular examinations performed to determine the species of *Trichinella* spp. responsible for this outbreak revealed the cause to be *T. spiralis*. Among the 214 cases were tourists and travelers from other countries such as Ireland and Germany. [40].

Furthermore, an outbreak in Germany in 2007 was caused by cured sausage from Romania. The epidemiological investigation revealed that *T. spiralis* was present in cured paprika sausage and streaky bacon made from a home-slaughtered pig in the form of minced meat, and these products had not been thoroughly heated. This outbreak is particularly important because it shows the significance of the imported meat products from countries where *Trichinella* spp. is present in domestic and sylvatic animals [41]. An outbreak of trichinellosis involving 107 people was reported in 2009 in Lithuania. Investigation performed during this outbreak revealed that homemade sausages from wild boar were the source of trichinellosis infection [42]. More recently, at the beginning of 2017, a trichinellosis outbreak occurred in France and Serbia due to meat from backyard pigs. In this outbreak, 20 cases of trichinellosis were reported, of which nine were in France and 11 in Serbia. The source of *Trichinella* spp. was a pork delicatessen in Serbia, from which products were also transferred to France. This outbreak shows that travelling to endemic regions is the simple way of acquiring trichinellosis. Therefore, travelers to endemic regions should be aware of a risk of consuming untested homemade raw meat products bought outside of official market in countries where the prevalence of trichinellosis is high [43].

The repeated occurrence of trichinellosis outbreaks among a group of friends and acquaintances indicates insufficient awareness of the risk of infection after the consumption of products containing raw or semi-raw meat products [42]. The meat from a single infected wild boar can contaminate a large batch of meat products and therefore can put hundreds of people at risk of trichinellosis if these products are made without proper heat treatment [44]. The cases of trichinellosis that get diagnosed in non-endemic countries are mostly related to infection while travelling or after illegal importation of meat products by travelers. Although the consumption of game meat is considered a healthy dietary habit, the incidental absence of meat inspection and low awareness of trichinellosis remain the cause of outbreaks of this disease [45].

## 5. Conclusions

The case described above indicates the need for better education on food safety among hunters and consumers. It also highlights the gaps in food control of meat derived from wild animals. The existing legal basis allows for taking the carcass of a wild boar from the hunting ground for one’s own needs. It seems there is an urgent need for changes at the local level that will prevent this type of practice. In areas endemic for *Trichinella*, it should be necessary to test for the presence of *Trichinella* in meat before handing over the boar, even if it is only for the hunter’s own needs.

## Figures and Tables

**Table 1:** The symptoms observed in cases of confirmed trichinellosis by age and gender (data obtained from Regional Sanitary Inspection in Poznan).

Age	Sex *	Symptoms				
		Fever	Muscle Pain	Swelling of the Eyelids	Abdominal Pain	Diarrhea
19	M	+	+	+		
19	M	+	+	+		
32	M	+	+	+	+	
33	M	+	+	+		
36	F	+	+	+		
39	F	+	+	+	+	
47	M	+	+	+		+
58	M	+	+	+	+	

* M—male; F—female.
